# Comparing the effects of self-selected MUsic versus predetermined music on patient ANXiety prior to gynaecological surgery: the MUANX randomized controlled trial

**DOI:** 10.1186/s13063-021-05511-2

**Published:** 2021-08-13

**Authors:** D. Reynaud, N. Bouscaren, V. Lenclume, M. Boukerrou

**Affiliations:** 1 Direction des Soins Infirmiers, de Rééducation et MédicoTechniques (Site Sud), CHU de la Réunion, 97410 Saint-Pierre, France; 2 Inserm CIC1410, CHU de la Réunion, Saint-Pierre, France; 3 Service de Gynécologie Obstétrique, PFME, CHU SUD de la Réunion, 350 97448 Saint-Pierre, BP France; 4 UFR santé de la Réunion, Université de la Réunion, Saint-Denis, France; 5 Centre d’étude périnatale de l’océan indien EA 7388, la Réunion, Saint Pierre, France

**Keywords:** Anxiety, Alternative and complementary therapy, Gynaecologic surgical procedures, Music therapy

## Abstract

**Background:**

Anxiety is frequently observed in the preoperative setting. The negative impact of preoperative anxiety is well known. In the context of gynaecological surgery, anxiety is exacerbated by the fact that the intervention can have catastrophic repercussions on a woman’s body image, sexuality, and psycho-affective well-being. Music listening is increasingly used as an alternative therapy for minimizing preoperative anxiety. Personal preferences, familiarity, and popularity may be key elements for an optimal relaxation response to music.

This study aimed to determine whether listening to self-selected music decreases preoperative anxiety in women scheduled to undergo gynaecologic surgery compared with predetermined music from an application (MUSIC CARE®).

**Methods:**

The MUANX study was a single-blind, monocentric, parallel, superiority, randomized controlled trial. A total of 174 women were included and randomized in two groups between August 2017 and September 2018. Patients in the intervention group listened to the personal music playlist that they had created before being hospitalized. Patients in the control group listened to the predetermined playlist on the MUSIC CARE® application. All patients received standard nursing care and listened to 20 min of music 1 h before surgery. Anxiety scores were assessed before and after the music session using Spielberger’s State-Trait Anxiety Inventory (STAI).

**Results:**

The mean age of the 171 evaluated patients was 41.5 years (SD = 10.0 years). Before the music session, the STAI state anxiety score was similar in the control group (M = 38.8, SD = 11.9) and the intervention group (M = 39.0, SD = 13.1). After the music session, this score had significantly decreased in both the control group (M = −7.2, SD = 9.0) and the intervention group (M = −5.5, SD = 6.6), with no significant difference in score reduction between groups. Physiological parameters were unchanged after the music session. No significant differences in postoperative measurements (pain intensity, hospitalization duration) were observed between the two groups.

**Conclusion:**

Self-selected music is as effective as predetermined music for reducing patient anxiety before gynaecological surgery. As it has no side effects and is easily applicable in gynaecological surgical services, this non-drug intervention may be proposed by healthcare professionals in the management of preoperative anxiety.

**Trial registration:**

The MUANX trial (MUsic therapy on ANXiety) is registered at the US National Institutes of Health (ClinicalTrials.gov) #NCT03226834. Registered on 24 July 2017. https://clinicaltrials.gov/ct2/show/NCT03226834?term=muanx&draw=2&rank=1

## Background

Surgery and preoperative waiting are anxiety-provoking situations [1, 2]. It is estimated that the prevalence of preoperative anxiety ranging from 25 to 80% depending on the type of surgery [3, 4]. Being in an unknown environment, the sense of losing control, perception of physical risk, dependence on strangers, separation from relatives, pre- and postoperative complications, and fear of mutilation, anaesthesia, and death are all factors that can trigger or aggravate preoperative anxiety [4–6].

This is especially the case in the context of gynaecological surgery, which most often results in the partial or total removal of an organ that is necessarily linked to maternity or femininity. Sexuality can be damaged [7]. For example, physical and psychological post-operative impacts of hysterectomy are real: hot flushes, night sweats, urinary incontinence, and urge incontinence [8]. The fear of pain, feeling of being rejected by their spouses, and sensation of mutilation were expressed by patients [9].The surgical management of gynaecological cancer can cause short- and long-term effects on sexuality, emotional well-being, reproductive function, and overall quality of life [10].

High levels of anxiety result in the activation of the autonomic nervous system and in negative physiological manifestations [2, 11–13]. These manifestations slow down the healing process, decrease the immune response, and increase the risk of infection, postoperative complications, pain, morbidity, and mortality [2, 4]. Preoperative anxiety might cause hemodynamic problems in the intraoperative period, increased analgesic need, and lower postoperative satisfaction of the patients in the postoperative period [14].

High levels of anxiety can complicate the administration of preoperative drugs, while negatively interfering with the induction of anaesthesia and delaying recovery [15].

In view of the above, reducing preoperative anxiety appears to be essential. Traditionally, the reduction of preoperative anxiety was based on the use of sedatives or anti-anxiety drugs administered prophylactically in the preoperative phase. However, these treatments can have negative side effects like drowsiness and respiratory depression. They can also interact with anaesthetic agents by prolonging the recovery phase and the duration of hospitalization [16]. In recent years, non-drug strategies have been developed to reduce preoperative anxiety. Acupuncture [17], hypnosis [18], non-pharmacological and non-prescription medicinal plant use [19], relaxation therapy [20], and Raj Yoga meditation [21] are among the techniques that have been shown to significantly reduce preoperative anxiety during hospitalization. Music therapy is one of these alternative strategies. A 2013 Cochrane systematic review concluded that music interventions can provide a viable alternative to sedatives and anti-anxiety drugs for the reduction of preoperative anxiety [2]. A common theory regarding the anxiety-reducing effects of music is that music can help patients focus their attention away from stressful events to something pleasant and soothing [2, 22].

In surgical context, listening to music has an effect on blood pressure and pulse rate, and the classic music resulted in lowered blood pressure and heart rate [23]. In addition, a 2014 randomized control trial found that anxiety scores and physiological parameters measured before gynaecological surgery were significantly lower in patients who had received music interventions than in those who had received standard care alone [24]. This study is the only we found in the context of gynaecological surgery.

To date, however, most published controlled randomized trials have evaluated the effects of music interventions on preoperative anxiety by comparing patients who listen to researcher-selected or predetermined music with patients who receive standard care alone. Few studies have compared the impact of different styles of music on preoperative anxiety [25]. This is unfortunate, as music may be perceived as relaxing or not depending on its intrinsic characteristics, which include complexities of tempo, harmony, rhythm, dynamic variations, and melody. Personal preferences, familiarity, and popularity may be key elements for an optimal relaxation response to music [25, 26]. Perceptions of music styles, rhythms, and sounds are known to vary depending on cultural background, ethnicity, and personal experience [27]. For example, Sega and Maloya are two popular music styles of Reunion Island, which is a French overseas territory with a multiethnic and multicultural population [28, 29]. These styles of music, also known as “Creole music”, are based on a traditional ensemble with African and Malagasy rhythmic influences.

Our study seeks to fill this gap in the literature by letting patients create their own, culturally adapted music playlist. Our hypothesis was that music therapy programmes, which use specific rhythm sequences to potentiate the anxiolytic effects of music, could be optimized by adapting them to the preferences and cultural contexts of patients.

The aim of our study was to determine whether listening to self-selected music decreases preoperative anxiety in women scheduled to undergo gynaecologic surgery as compared with predetermined music from a music therapy software programme (MUSIC CARE®). Physiological parameter evolutions after the music session, post-operative pain intensity, and duration of post-surgical hospitalization were also compared.

## Methods

Complete information on research methods may be found in study protocol, which is freely available at https://link.springer.com/article/10.1186/s13063-018-3093-6 [27].

### Trial design

The MUANX study was a single-blind, monocentric, parallel, superiority, randomized controlled trial conducted in the gynaecological surgery department in French hospital. Patients were randomly allocated to one of two groups: the intervention group listened to a self-selected playlist and the control group listened to a predetermined playlist selected from among those available on the MUSIC CARE® application. Healthcare team members were blinded to patient allocation.

### Participants

The criteria for inclusion in the study were being aged 18 to 70 years; being scheduled to undergo gynaecological surgery under general anaesthesia or spinal anaesthesia at the gynaecological surgery department of the South Reunion Island University Hospital between August 2017 and September 2018; and not receiving anti-anxiety drugs prior to surgery. Exclusion criteria were having generalized anxiety disorder, depression, dementia, or neuromotor disabilities. All eligible patients received standard nursing care and were asked to create a 20-min music playlist based on their personal tastes before being hospitalized.

Eligible adults were informed about this study by gynaecologists during their operative consultation or anaesthesiologists during pre-operative screening.

### Interventions

#### Intervention group: self-selected music group

Patients in the intervention group listened to the personal music playlist that they had created before being hospitalized. They were allowed to listen to the playlist on the device of their choice: smartphone, tablet, computer, or CD player.

#### Control group: predetermined music group

Patients in the control group listened to the predetermined playlist that they had selected before randomization from among those available on the MUSIC CARE® application [30]. MUSIC CARE® is a non-drug alternative therapy commonly used for the management of anxiety and pain. This standardized music therapy application relies on the U sequence, a music relaxation technique based on the principles of hypno-analgesia. The U sequence is composed of three phases: (1) a sleep-inducing phase (stimulating rhythms), which corresponds to the patient’s current state of consciousness; (2) a relaxation phase (slow rhythms); and (3) an awakening phase (moderate rhythms), which signals the end of the session.

The objective of MUSIC CARE® is to promote wellbeing and to induce a relaxed state in patients by loosening their muscles and reducing their level of anxiety or pain. Patients were able to choose from among 25 MUSIC CARE® playlists, which include reverie, Cuban night, guitar ballad, film scores, folk guitar, etc. Sega and Maloya were not included in this playlist.

A department nurse or care provider with training in MUSIC CARE® was in charge of explaining the application to study participants.

#### Intervention protocol

Each patient was asked to create a personal music playlist based on her personal tastes (before being hospitalized) and to choose a predetermined playlist from those available on the MUSIC CARE® application (before randomization).

The music intervention started within the hour preceding surgery. The planned duration of the music session was 20 min [2]. To ensure the blindness of care team members, a trained member of the research team brought a MUSIC CARE® tablet to each patient (even to patients in the intervention group). Each patient was notified of the group to which she had been allocated and then was informed that she could start listening to the music playlist as determined by randomization: either the predetermined playlist that she had selected from the MUSIC CARE® application (on the provided tablet), or the playlist that she had created (on the device of her choice). After 20 min, the research team member returned to inform the patient that the session was over and recovered the equipment. During the music session, patients were alone in their individual room, seated in an armchair or lying in the supine position. They were not disturbed by anyone. Patients were reminded that they could listen only to the predetermined playlist or to the self-selected playlist (and to nothing else), as determined by randomization. There were no restrictions on volume levels [27].

### Outcomes and other variables of interest

#### Primary outcome

The primary outcome was the difference between the preoperative anxiety score assessed 15 to 20 min before the music-listening session and the preoperative anxiety score assessed straight after the session, as measured with the STAI (State-Trait Anxiety Inventory) for state anxiety. The mean change score from baseline to post music intervention was used to compare groups. The state anxiety score was measured using the second part of the Spielberger’s STAI questionnaire (French version: *STAI form Y-A*).

The STAI is a validated questionnaire that is widely used to measure patient anxiety in the context of music therapy [31, 32]. This self-evaluation questionnaire consists of 40 statements, each describing an attitude. It is composed of two separate sections of 20 statements each: The first part assesses trait anxiety and the second measures state anxiety. Total scores for trait and state anxiety range between 20 and 80: the higher the score, the more the patient experiences (state and trait) anxiety. Classifications into five levels of anxiety were made: greater than 65 (very high), 56 to 65 (high), 46 to 55 (medium), 36 to 45 (low), and less than 35 (very low).

The State-Trait Anxiety Inventory can be used in the evaluation of preoperative anxiety among patients scheduled for surgery [33].

#### Secondary outcomes

The secondary outcomes were as follows: (1) the difference in the Visual Analogue Scale (VAS) anxiety score between before and after the music session [34]. The VAS test was administered 15 to 20 min before the start of the music-listening session and after STAI test, and a second time shortly after the music session ended and STAI test; (2) the difference in physiological parameters (pulse rate and blood pressure). A nurse recorded parameters 15 to 20 min before the session, after she administered the STAI and VAS tests, and did so again shortly after the session, after she administers the STAI and VAS tests a second time and before the patient went to the operating room; (3) the postoperative pain intensity score, as measured with a Numeric Rating Scale (NRS) [35] 30 min after returning from the operating room; and (4) the duration of hospitalization from admission to discharge, recorded on the day of discharge.

#### Complementary measures of patient anxiety

The trait anxiety score was measured based on the answers given by study participants to the first part of the STAI questionnaire (French version: STAI form Y-B). The latter was filled during the inclusion visit one day before surgery.

### Sample size

The 2013 Cochrane systematic review found that music listening resulted, on average, in a 6-point decrease in the preoperative STAI state anxiety score of patients compared to standard care alone [2]. Our study hypothesis was that patients who listened to self-selected music would show a greater decrease in the STAI state anxiety score than those who listened to predetermined music. Assuming an intra-individual correlation of 0.5 between the STAI state anxiety score before and after the music session (conservative assumption), the inclusion of 85 patients in each group (170 in total) was expected to yield a significant difference in the reduction of the STAI state anxiety score between the predetermined music group (6-point decrease) and the self-selected music group (9-point decrease), with an alpha level of 5% and a statistical power of 80%. The expected 3-point difference in score reduction was considered to be clinically relevant.

### Randomization

#### Sequence production

The randomization of eligible patients was carried out on the day of surgery using Ennov Clinical®. Randomization was single-blinded and balanced between the control group and the intervention group, with a 1:1 allocation ratio and without stratification. Patients were aware of the group to which they had been allocated, but care team members were not. The minimization method programmed in the CSRandomization module of Ennov Clinical® was used to allocate patients. The predictive side of this method was reduced with the two tools available in the CSRandomization module [35, 36].

#### Blinding

On the day of surgery, randomization was performed by the research team interviewer directly via the electronic case report form (eCRF). Standard care providers and care team members in charge of outcome assessment were blinded to patients’ group allocation. Only research team members were aware of the group to which patients had been allocated. ‘Do Not Disturb’ signs were placed on patient room doors during the music session. Patients were warned not to mention their group allocation to care team members.

#### Statistical methods

Data were expressed as *n* (percentages) or means, standard deviation (SD). The two groups were compared using the Mann-Whitney *U* test for quantitative variables and the chi-square test or Fisher’s exact test or the Fisher-Freeman-Halton test for categorical variables, as appropriate. Measures before and after the music session were compared using the Wilcoxon signed-rank tests. The evolution of the STAI state anxiety score was evaluated according to the STAI trait anxiety score level using the Kruskal-Wallis test. Multiple 2 to 2 comparisons of the different components of the STAI trait anxiety score were performed using the Mann-Whitney *U* test with an alpha level of 0.83% (Bonferroni correction: 5%/6). Spearman’s correlation coefficient between the STAI state anxiety score and the STAI trait anxiety score was calculated.

One patient with missing data on the primary outcome was excluded from the analysis. Except for the multiple 2 to 2 comparisons, the significance level was set to 5%. Analyses were performed using SAS® version 9.4 (SAS Institute Inc., Cary, NC, USA).

## Results

### Recruitment and flow of participants

**Fig. 1:**
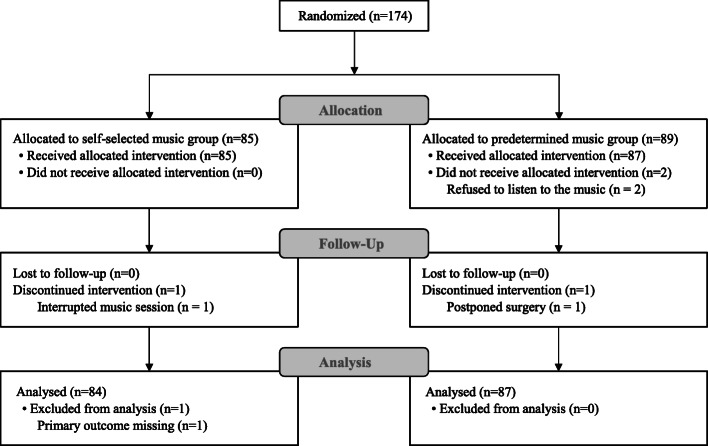
Flow chart of participant selection

A total of 174 women were included and randomized in the MUANX trial between 1 August 2017 and 20 September 2018. Of these, 89 (51%) were allocated to the control group (predetermined music group) and 85 (49%) to the intervention group (self-selected music group). In the control group, two patients were excluded because they refused to listen to music. One patient had postponed surgery after she received music therapy. For this patient, we analysed the primary outcome evaluation (but data were missing on postoperative measurements). In the self-selected music group, one patient was excluded due to missing data on the primary outcome and one patient included in analysis had an interruption during the music listening. The flow chart describing the process of participant selection is presented in Fig. 1. Overall, 87 (51%) patients from the predetermined music group and 84 (49%) patients from the self-selected music group were analysed.

### Baseline data

Baseline socio-demographic characteristics, musical habits, medical history, and types of surgery were similar in the two groups (Table 1). Women were aged 41.5 years (SD = 10.0 years). As regards musical habits, 116 (68.2%) patients declared that they listened to music several times a day and 17 (9.9%) reported playing an instrument. The preferred musical styles were popular music (69.6%, *n* = 119) and world music (61.4%, *n* = 105). As regards medical history, 143 (84.6%) women had a history of hospitalization, 136 (81.4%) had a history of anaesthesia, 133 (78.2%) had a history of surgery, and 145 (84.8%) had a history of pregnancy. During surgery, 170 (99.6%) had general anaesthesia and 1 (0.6%) had spinal anaesthesia. The patients were mainly operated for uterine surgery (63.2%, *n* = 108). Baseline socio-demographic characteristics, musical habits, medical history, and types of surgery are shown in Table 1.

**Table 1:** Baseline characteristics of the total sample, the self-selected music group, and the predetermined music group

	Total sample (***n*** = 171) n (%) or mean, SD	Self-selected music group (***n*** = 84) n (%) or mean, SD	Predetermined music group (***n*** = 87) *n* (%) or mean, SD	***p***-value
**Socio-demographic characteristics**				
Age (years) [mean, SD]^a^	41.5, 10.0	42.1, 10.1	41.1, 10.0	0.363
Current employment (1 MD)	85 (50.0)	48 (57.1)	37 (43.0)	0.066
Education level				
Primary education or less	45 (26.3)	19 (22.6)	26 (29.9)	0.550
Secondary education	63 (36.8)	33 (39.3)	30 (34.5)	
A-level or higher education	63 (36.8)	32 (38.1)	31 (35.6)	
**Musical habits**				
Play of an instrument	17 (9.9)	7 (8.3)	10 (11.5)	0.490
Preferred musical style				
Classical music	82 (48.0)	42 (50.0)	40 (46.0)	0.599
World music	105 (61.4)	49 (58.3)	56 (64.4)	0.418
Jazz	60 (35.1)	31 (36.9)	29 (33.3)	0.625
Popular music	119 (69.6)	58 (69.0)	61 (70.1)	0.879
Modern music	55 (32.2)	26 (31.0)	29 (33.3)	0.739
Creole music	41 (24.0)	20 (23.8)	21 (24.1)	0.960
Hymn music	18 (10.5)	9 (10.7)	9 (10.3)	0.937
Relaxation music	8 (4.7)	2 (2.4)	6 (6.9)	0.278
Other music	31 (18.1)	14 (16.7)	17 (19.5)	0.626
Music listening frequency^c^ (1 MD)				
Once a week	10 (5.9)	2 (2.4)	8 (9.3)	0.267
A few times a week	36 (21.2)	18 (21.4)	18 (20.9)	
More than once a day	8 (4.7)	5 (6.0)	3 (3.5)	
Several times a day	116 (68.2)	59 (70.2)	57 (66.3)	
**Medical history**				
History of hospitalization (2 MD)	143 (84.6)	69 (83.1)	74 (86.0)	0.600
History of anaesthesia (4 MD)	136 (81.4)	67 (82.7)	69 (80.2)	0.680
History of surgery (1 MD)	133 (78.2)	63 (75.9)	70 (80.5)	0.472
History of pregnancy	145 (84.8)	74 (88.1)	71 (81.6)	0.238
**Type of surgery**				
Tubal and ovarian	22 (12.9)	10 (11.9)	12 (13.8)	0.825
Uterine	108 (63.2)	52 (61.9)	56 (64.4)	
Breast	30 (17.5)	17 (20.2)	13 (14.9)	
Others	11 (6.4)	5 (6.0)	6 (6.9)	

*MD* missing data

Data are *n* (percentages) or means, SD. All *p* values refer to comparisons between the two groups using the Mann-Whitney *U* test (^a^) for quantitative variables and the chi-square test or Fisher’s exact test (^b^) or Fisher-Freeman-Halton test (^c^) for categorical variables

### Initial anxiety scores and physiological parameters

Before the music session, the mean STAI state anxiety score for the total sample was 38.9 (SD = 12.4). The initial STAI state anxiety score was similar in the control group and the intervention group (M = 38.8, SD = 11.9 vs. M = 39.0, SD = 13.1, *p* = 0.857). Nineteen (11.1%) women had a high/very high initial STAI state anxiety score (≥ 56). The initial mean VAS anxiety score for the total sample was 3.4, SD = 2.5 (M = 3.2, SD = 2.6 in control group vs. M = 3.6, SD = 2.4 in intervention group, *p* = 0.209). As regards physiological parameters, 53 (31%) patients had a high initial pulse rate and 37 (21.8%) had a high initial blood pressure. No significant differences in initial anxiety scores or initial physiological parameters were found between the two groups (*p* > 0.05). Anxiety scores and physiological parameters before the music session are presented in Table 2.

**Table 2:** Anxiety scores and physiological parameters of patients before the music session

		Total sample (***n*** = 171) *n* (%) or mean, SD	Self-selected music group (***n*** = 84) *n* (%) or mean, SD	Predetermined music group (***n*** = 87) *n* (%) or mean, SD	***p***-value
**Anxiety scores**					
STAI trait anxiety score (STAI form Y-B) (1 MD)	Anxiety score^a^	42.5, 9.8	42.0, 10.0	43.1, 9.6	0.309
	Very low (≤ 35)	42 (24.7)	23 (27.4)	19 (22.1)	0.298
	Low (36–45)	67 (39.4)	36 (42.9)	31 (36.1)	
	Middle (46–55)	41 (24.1)	15 (17.8)	26 (30.2)	
	High (56–65)/very high (> 65)	20 (11.8)	10 (11.9)	10 (11.6)	
STAI state anxiety score (STAI form Y-A)	Anxiety score^a^	38.9, 12.4	39.0, 13.1	38.8, 11.9	0.857
	Very low (≤ 35)	78 (45.6)	40 (47.6)	38 (43.7)	0.248
	Low (36–45)	45 (26.3)	22 (26.2)	23 (26.4)	
	Middle (46–55)	29 (17.0)	10 (11.9)	19 (21.8)	
	High (56–65)/very high (> 65)	19 (11.1)	12 (14.3)	7 (8.1)	
VAS anxiety score (2 MD)	Anxiety score^a^	3.4, 2.5	3.6, 2.4	3.2, 2.6	0.209
**Physiological parameters**					
Pulse rate	Rate (Bpm)^a^	75.6, 13.1	74.2, 13.1	76.9, 13.0	0.085
	> 80 Bpm	53 (31.0)	26 (31.0)	27 (31.0)	0.991
Systolic blood pressure	Pressure (mmHg)^a^	121.4, 17.6	120.5, 15.4	122.3, 19.5	0.493
	≥ 140 mmHg	27 (15.8)	12 (14.3)	15 (17.2)	0.596
Diastolic blood pressure (1 MD)	Pressure (mmHg)^a^	75.0, 12.4	74.0, 11.8	76, 13.0	0.325
	≥ 90 mmHg	27 (15.9)	9 (10.8)	18 (20.7)	0.079
Hypertension (1 MD)	≥ 140/90 mmHg	37 (21.8)	15 (18.1)	22 (25.3)	0.255

*MD* missing data

Data are *n* (percentages) or means, SD. All *p*-values refer to comparisons between the two groups using the Mann-Whitney *U* test (^a^) for quantitative variables and the chi-square test for categorical variables

### Music session duration and surgical procedure duration

No significant difference in music session duration was found between the two groups (M = 19.8, SD = 1.5 min in the predetermined music group vs. M = 19.9, SD = 1.7 min in the self-selected music group, *p* = 0.160). Likewise, no significant difference in surgical procedure duration was observed between the two groups (M = 170.1, SD = 87.7 min in the predetermined music group vs. M = 183.6, SD = 103.1 min in the self-selected music group, *p* = 0.420). Surgery was initiated 92.2 min (SD = 60.1 min) after the music session had ended, with no significant difference between the two groups (*p* = 0.286).

### Outcomes

#### Primary outcome

After the music session, a significant decrease in the STAI state anxiety score (M = −6.4, SD = 8.0, *p* < 0.001) was observed in the total sample. The STAI state anxiety score had also decreased significantly between before and after the music session in each group (M = −7.2, SD = 9.0, *p* < 0.001 in the predetermined music group vs. M = −5.5, SD = 6.6, p < 0.001 in the self-selected music group). There was no significant difference in score reduction between the two groups (*p* = 0.215). The evolution of the STAI state anxiety score between before and after the music session is shown in Table 3.

**Table 3:** Evolution of anxiety scores, evolution of physiological parameters, and postoperative measurements

	Total sample (***n*** = 171) *n* (%) or mean, SD	Self-selected music group (***n*** = 84) *n* (%) or mean, SD	Predetermined music group (***n*** = 87) *n* (%) or mean, SD	***p-***value
**Evolutions of anxiety scores**				
STAI state anxiety score (STAI form Y-A)	−6.4, 8.0	−5.5, 6.6	−7.2, 9.0	0.215
VAS anxiety score (4 MD)	−1.1, 1.9	−1.1, 1.8	−1.1, 2.0	0.798
**Evolutions of physiological parameters**				
Pulse rate (bpm) (2 MD)	−1.5, 12.5	−0.9, 11.1	−2.1, 13.8	0.661
Systolic blood pressure (mmHg) (2 MD)	0.4, 14.3	0.8, 15.5	−0.0, 13.2	0.466
Diastolic blood pressure (mmHg) (3 MD)	1.3, 12.8	2.7, 11.9	−0.2, 13.5	0.213
**Postoperative measurements**				
Pain intensity score (Numerical Rating Scale)	2.5, 2.5	2.5, 2.7	2.5, 2.3	0.480
Duration of hospitalization (days)	3.6, 1.7	3.5, 1.8	3.7, 1.6	0.250

*MD* missing data

Data are means, SD. All *p*-values refer to comparisons between the two groups using the Mann-Whitney *U* test

#### Secondary outcomes

After the music session, a significant decrease in the VAS anxiety score (M = −1.1, SD = 1.9, *p* < 0.001) was observed in the total sample. The VAS anxiety score had also decreased significantly in each group (M = −1.1, SD = 2.0, *p* < 0.001 in the predetermined music group vs. M = −1.1, SD = 1.8, *p* < 0.001 in the self-selected music group). There was no significant difference in score reduction between the two groups (*p* = 0.798). The evolution of the VAS anxiety score between before and after the music session is shown in Table 3.

No significant reduction in pulse rate (M = −1.5, SD = 12.5 Bpm, *p* = 0.170), systolic blood pressure (M = 0.4, SD = 14.3 mmHg, *p* = 0.804), or diastolic blood pressure (M = 1.3, SD = 12.8 mmHg, *p* = 0.169) was observed in the total sample. There was no significant difference in the reduction of physiological parameters between the two groups (*p* > 0.05). The evolution of physiological parameters between before and after the music session is shown in Table 3.

No significant difference in the postoperative pain intensity score was observed between the two groups (*p* = 0.480). Likewise, no significant difference in the duration of hospitalization was found between the two groups (*p* = 0.250). The mean postoperative pain intensity score was 2.5 (SD = 2.5) and the mean duration of hospitalization was 3.6 days (SD = 1.7 days). Postoperative measurements are shown in Table 3.

#### Complementary measure of patient anxiety

Participants had a mean STAI trait anxiety score of 42.5 (SD = 9.8). Twenty (11.8%) women had a high/very high STAI trait anxiety score (≥ 56).

### Ancillary analyses

#### Correlation between the STAI state anxiety score and the STAI trait anxiety score

**Fig. 2:**
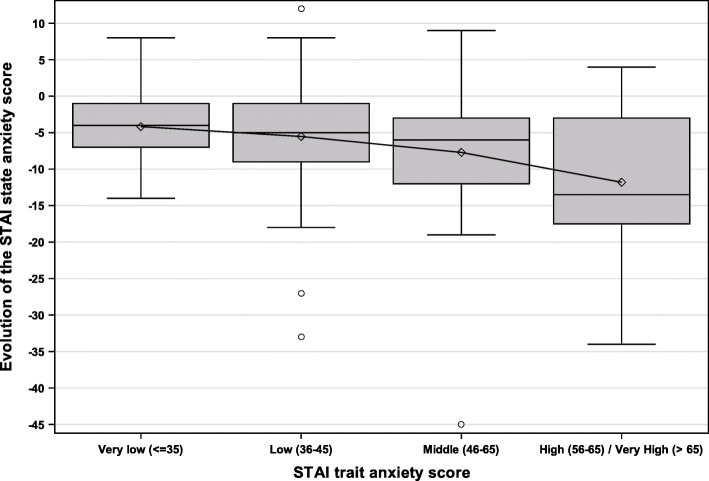
Evolution of the STAI state anxiety score according to the STAI trait anxiety score

The STAI state anxiety score before the music session was correlated to the STAI trait anxiety score (Rho = 0.46, *p* < 0.001). The reduction of the STAI state anxiety score between before and after the music session was inversely correlated to the STAI trait anxiety score (Rho = −0.24, *p* = 0.001). On average, the STAI state anxiety score decreased by 4.2 points (SD = 4.7 points) in patients with a very low STAI trait anxiety score (≤ 35), by 5.5 (SD = 7.6 points) in patients with a low STAI trait anxiety score (36–45), by 7.7 points (SD = 8.6 points) in patients with a middle STAI trait anxiety score (46–55), and by 11.8 (SD = 10.1 points) in patients with a high/very high STAI trait anxiety score (≥ 56) (*p* = 0.012). Comparisons 2 to 2 showed a significant difference in the reduction of the STAI state anxiety score between patients with a very low STAI trait anxiety score and patients with a high/very high STAI trait anxiety score (*p* = 0.003). The evolution of the STAI state anxiety score according to the STAI trait anxiety score is shown in Fig. 2.

#### Effectiveness of local music

In the self-selected music group, 23 (27.4%) patients had included local music in their personal playlist. After the music session, the STAI state anxiety score had decreased by 7.4 points (SD = 7.4 points) in these patients, compared with a decrease of 4.8 (SD = 6.2 points) in the 61 (72.6%) patients who had not included local music in their personal playlist (*p* = 0.154). The decrease in the STAI state anxiety score observed in patients who had included local music in their personal playlist was not significantly different from the decrease observed in patients who listened to predetermined music (M = −7.4, SD = 7.4 vs. M = −7.2, SD = 9.0, *p* = 0.763).

### Adverse events

One adverse event was reported in our study. However, it was not imputable to the study but to a sensory and motor deficit that developed in a patient on return from the operating room.

## Discussion

Feelings of insecurity, the sense of losing control, fear of death, and pre- and postoperative complications have all been shown to increase the anxiety levels and physiological parameters of patients prior to surgery [1, 5, 6]. In the context of gynaecological surgery, preoperative anxiety is increased by the fact that the intervention can have catastrophic repercussions on a woman’s body image, sexuality, and psycho-affective well-being. In view of this, non-drug interventions aimed at relaxation are increasingly proposed to patients during the preoperative phase [7, 8, 10]. Music therapy is one such intervention.

The aim of our study was to determine whether listening to self-selected music decreases anxiety in women scheduled to undergo gynaecologic surgery, as compared with predetermined music from a music therapy application (MUSIC CARE®). Our main finding is that preoperative anxiety scores, between after and before music session, decrease significantly in patients who receive music therapy, regardless of whether they listen to self-selected music or predetermined music. No significant difference in anxiety reduction was observed between the predetermined music group and the self-selected music group. By contrast, physiological parameters (pulse rate and blood pressure) had not decreased in either group after the music session. Our finding did not confirm the research hypothesis as measured by the primary outcome. We can consider the quality of music playlist created by patients. In fact, several factors are important for an anxiolytic and positive effect of music on anxiety: a high degree of periodicity, a catch melody line, a key that is experience as pleasant, few changes in volume or rhythm, harmony sequences that are rousing, and an absence of song words [26]. Each patient was asked to create a personal music playlist only based on her personal tastes before being hospitalized. Professional healthcare (nurses and doctors) should have given them a clearer explanation to create a relevant playlist in line with the knowledge on anxiolytic effects of music. In our study, in fact, the playlists made by the patients did not comply with the abovementioned criteria, which in contrast is the case with MUSIC CARE. The supposedly superior anxiolytic effect produced essentially by the musical preferences could not be achieved by not meeting the criteria.

Another possible reason for these results is that 80% patients included in the study has history of anaesthesia and history of surgery, so the patients are experienced in hospital and then had lower level of anxiety. The baseline STAY score in our study was 38.9 ± 12.4 points whereas the average pre-surgical score found in the literature is 43 ± 8 points [2].

Our results are in line with the published literature, including the 2013 Cochrane systematic review which found that music listening can have a beneficial effect on preoperative anxiety [2, 24, 25, 37]. However, they do not confirm our hypothesis that anxiety reduction would be greater in patients who listened to self-selected music compared to those who listened to predetermined music. Still, our study shows that self-selected music is as effective as predetermined music for reducing the anxiety of women scheduled to undergo gynaecological surgery. This finding suggests that the cost of music therapy can be minimized by allowing patients to listen to their own self-selected playlists before undergoing surgery.

In our study, we expected that patients who listened to self-selected music would have included local music, also Creole music (Sega and Maloya) [28, 29], in their personal playlist. Only 23 (27.4%) of patients in the intervention group, all of whom were born in Reunion Island, recorded and listened to Sega or Maloya before surgery. The STAI state anxiety score of these patients was found to have decreased after the music session. However, no significant difference in score reduction was observed between them and patients who did not include local music in their personal playlist. And yet, music listening based on individual preferences have been shown to impact the evolution of patient anxiety. Thus, a 2018 Turkish study found that music therapy using local music was more effective compared to music therapy using others kinds of music. Likewise, a 2018 German study analysed the effects on health of different musical styles (classical music, meditation music, rock, heavy metal, Latin American music). It found that the musical styles with the strongest anxiolytic power were classical music and meditation music [26].

It should be noted that our patients did not have high levels of anxiety prior to the music session. Indeed, only 19 (11.1%) of our patients showed high/very high STAI state anxiety scores at the beginning of the study. On the one hand, these results confirm those of a 2012 study on the prevalence of anxiety in patients scheduled to undergo gynaecological surgery [38]. On the other, they contradict the findings of a 2018 studies, in which between 25 and 80% of patients scheduled to undergo surgery of all types had high STAI state anxiety scores [3, 4]. The lower anxiety levels observed in gynaecological patients may be explained by the fact that these are given adequate psychological preparation before undergoing surgery [39]. While physicians inform patients about the type of therapy considered (along with its expected risks and benefits) to help them make an informed decision, nurses provide patients with the information they need to cope with stressful situations. Gynaecological nurses clearly have an important role to play in the area of anxiety reduction and are therefore best positioned to propose music therapy to their patients. In our study, patients had history of anaesthesia and history of surgery, so they are experienced in hospital. These factors can also explain the low level of anxiety in our population.

Our study has several limitations that must be acknowledged. First, all patients were women. This may have led to a selection bias, which makes it difficult to extrapolate our results to the entire surgical population. Indeed, our study focused on gynaecological surgery; it might be interesting to extend it to other types of surgery and in a male population. Second, our study was single-blind: while care team members were unaware of the group to which patients had been allocated, patients themselves were evidently aware of this. Third, all patients in the control group used the provided headphones to listen to the predetermined playlist, whereas patients in the intervention group were allowed to listen to their personal playlist without headphones if they wished. Our study did not explore the differential effects of music listening on the anxiety of patients depending on whether or not they used headphones. However, it should be noted that a 2011 study found no difference in the efficacy of music therapy between patients who listened to music on headphones and patients who listened to broadcast music [40].

## Conclusion

Our study shows that listening to self-selected music is as effective as listening to predetermined music for reducing patient anxiety prior to gynaecological surgery. A 20-min music session was found to significantly reduce anxiety in gynaecological patients. Music listening is a simple anti-anxiety therapy that has no side effects and that can be proposed by nurses in the gynaecological preoperative context to complement classical therapy.

## Data Availability

The datasets generated and analysed during the current study are available from the corresponding author on reasonable request.

## Abbreviations

CRF: Case report form
NRS: Numerical Rating Scale
RCT: Randomized controlled trial
STAI: State-Trait Anxiety Inventory
VAS: Visual Analogue Scale

## References

[1] Celik F, Edipoglu IS. Evaluation of preoperative anxiety and fear of anesthesia using APAIS score. Eur J Med Res. 2018;23(1):41. 10.1186/s40001-018-0339-4 PMC6131845 30205837

[2] Bradt J, Dileo C, Shim M (2013). Music interventions for preoperative anxiety. Cochrane Database Syst Rev..

[3] Aust H, Eberhart L, Sturm T, Schuster M, Nestoriuc Y, Brehm F, et al. A cross-sectional study on preoperative anxiety in adults. J Psychosom Res. 1 août 2018;111:133-9. 10.1016/j.jpsychores.2018.05.012 29935747

[4] Stamenkovic DM, Rancic NK, Latas MB, Neskovic V, Rondovic GM, Wu JD, Cattano D (2018). Preoperative anxiety and implications on postoperative recovery: what can we do to change our history. Minerva Anestesiol..

[5] Eberhart L, Aust H, Schuster M, Sturm T, Gehling M, Euteneuer F, et al. Preoperative anxiety in adults - a cross-sectional study on specific fears and risk factors. BMC Psychiatry [Internet]. 30 mars 2020 [cité 23 juill 2020];20. Disponible sur: https://www.ncbi.nlm.nih.gov/pmc/articles/PMC7106568/10.1186/s12888-020-02552-w PMC7106568 32228525

[6] Gonçalves KKN, Da Silva JI, Gomes ET, De Souza Pinheiro LL, Figueiredo TR, Da Silva Bezerra SMM. Anxiety in the preoperative period of heart surgery. Rev Bras Enferm. 2016;69(2):397-403. 10.1590/0034-7167.2016690225i. 10.1590/0034-7167.2016690225i 27280578

[7] Pauls RN (2010). Impact of gynecological surgery on female sexual function. Int J Impot Res. avr.

[8] Kołodziejczyk A, Pawłowski T (2019). Negative body image in breast cancer patients. Adv Clin Exp Med Off Organ Wroclaw Med Univ. août.

[9] Gueye M, Diouf AA, Cisse A, Coulbary AS, Moreau JC, Diouf A (2014). Consequences of hysterectomy at the national-hospital of Pikine in Dakar. Tunis Med..

[10] Stabile C, Gunn A, Sonoda Y, Carter J (2015). Emotional and sexual concerns in women undergoing pelvic surgery and associated treatment for gynecologic cancer. Transl Androl Urol..

[11] Shevde K, Panagopoulos G (1991). A survey of 800 patients’ knowledge, attitudes, and concerns regarding anesthesia. Anesth Analg. août.

[12] Mackenzie JW (1989). Daycase anaesthesia and anxiety. A study of anxiety profiles amongst patients attending a day bed unit. Anaesthesia. mai.

[13] Cooke M, Chaboyer W, Schluter P, Hiratos M (2005). The effect of music on preoperative anxiety in day surgery. J Adv Nurs. oct.

[14] Bayrak A, Sagiroglu G, Copuroglu E (2019). Effects of Preoperative Anxiety on Intraoperative Hemodynamics and Postoperative Pain. J Coll Physicians Surg--Pak JCPSP. sept.

[15] Ahmetovic-Djug J, Hasukic S, Djug H, Hasukic B, Jahic A (2017). Impact of preoperative anxiety in patients on hemodynamic changes and a dose of anesthetic during induction of anesthesia. Med Arch..

[16] Agarwal A, Ranjan R, Dhiraaj S, Lakra A, Kumar M, Singh U (2005). Acupressure for prevention of pre-operative anxiety: a prospective, randomised, placebo controlled study. Anaesthesia. oct.

[17] Kwon C-Y, Lee B. Acupuncture or acupressure on Yintang (EX-HN 3) for anxiety: a preliminary review. Med Acupunct. 1 avr 2018;30(2):73-9. 10.1089/acu.2017.1268 PMC5908420 29682147

[18] Akgul A, Guner B, Çırak M, Çelik D, Hergünsel O, Bedirhan S (2016). The beneficial effect of hypnosis in elective cardiac surgery: a preliminary study. Thorac Cardiovasc Surg. oct.

[19] Akhlaghi M, Shabanian G, Rafieian-Kopaei M, Parvin N, Saadat M, Akhlaghi M (2011). Citrus aurantium blossom and preoperative anxiety. Rev Bras Anestesiol. déc.

[20] Kim H-S, Kim EJ (2018). Effects of relaxation therapy on anxiety disorders: a systematic review and meta-analysis. Arch Psychiatr Nurs. avr.

[21] Kiran U, Ladha S, Makhija N, Kapoor PM, Choudhury M, Das S, Gharde P, Malik V, Airan B (2017). The role of Rajyoga meditation for modulation of anxiety and serum cortisol in patients undergoing coronary artery bypass surgery: a prospective randomized control study. Ann Card Anaesth. juin.

[22] de Witte M, Spruit A, van Hooren S, Moonen X, Stams G-J. Effects of music interventions on stress-related outcomes: a systematic review and two meta-analyses. Health Psychol Rev. 2 avr 2020;14(2):294-324. 10.1080/17437199.2019.1627897 31167611

[23] Trappe H-J, Voit G (2016). The cardiovascular effect of musical genres. Dtsch Arztebl Int..

[24] Labrague LJ, McEnroe-Petitte DM. Influence of music on preoperative anxiety and physiologic parameters in women undergoing gynecologic surgery. Clin Nurs Res. 1 avr 2016;25(2):157-73. 10.1177/1054773814544168 25078946

[25] Uğraş GA, Yıldırım G, Yüksel S, Öztürkçü Y, Kuzdere M, Öztekin SD (2018). The effect of different types of music on patients’ preoperative anxiety: a randomized controlled trial. Complement Ther Clin Pract. mai.

[26] Tan X, Yowler CJ, Super DM, Fratianne RB (2012). The interplay of preference, familiarity and psychophysical properties in defining relaxation music. J Music Ther..

[27] Petot T, Bouscaren N, Maillard O, Huiart L, Boukerrou M, Reynaud D. Comparing the effects of self-selected music versus predetermined music on patient anxiety prior to gynaecological surgery: a study protocol for a randomised controlled trial. Trials. 7 janv 2019;20(1):20. 10.1186/s13063-018-3093-6 PMC6323656 30616674

[28] Desrosiers B. Ile de la Réunion: musiques et identité. MC.1992;20 Available from:https://journals.lib.unb.ca/index.php/MC/article/view/21711

[29] Samson G (2011). Le maloya au patrimoine de l'humanité. Enjeux culturels, politiques et éthiques d'une labellisation. Cahiers d'ethnomusicologie.

[30] Guétin S, Diego E de, Mohy F, Adolphe C, Hoareau G, Touchon J, et al. A patient-controlled, smartphone-based music intervention to reduce pain—a multi-center observational study of patients with chronic pain. Eur J Integr Med. 1 juin 2016;8(3):182-7.

[31] Gauthier J, Bouchard S (1993). Adaptation canadienne-française de la forme révisée du State–Trait Anxiety Inventory de Spielberger. [A French-Canadian adaptation of the revised version of Spielberger’s State–Trait Anxiety Inventory.]. Can J Behav Sci Rev Can Sci Comport..

[32] Spielberger CD (1983). Manual for the State-Trait Anxiety Inventory STAI (form Y)(« self-evaluation questionnaire »).

[33] Zemła AJ, Nowicka-Sauer K, Jarmoszewicz K, Wera K, Batkiewicz S, Pietrzykowska M (2019). Measures of preoperative anxiety. Anaesthesiol Intensive Ther..

[34] Hjermstad MJ, Fayers PM, Haugen DF, Caraceni A, Hanks GW, Loge JH, Fainsinger R, Aass N, Kaasa S, European Palliative Care Research Collaborative (EPCRC). Studies comparing Numerical Rating Scales, Verbal Rating Scales, and Visual Analogue Scales for assessment of pain intensity in adults: a systematic literature review. J Pain Symptom Manage. 2011;41(6):1073-93. 10.1016/j.jpainsymman.2010.08.016. 10.1016/j.jpainsymman.2010.08.016 21621130

[35] Moher D, Hopewell S, Schulz KF, Montori V, Gøtzsche PC, Devereaux PJ, et al. CONSORT 2010 explanation and elaboration: updated guidelines for reporting parallel group randomised trials. BMJ. 2010;340:c869. 10.1136/bmj.c869 PMC2844943 20332511

[36] Scott NW, McPherson GC, Ramsay CR (2002). Campbell MK. The method of minimization for allocation to clinical trials. a review. Control Clin Trials. déc.

[37] Kovac M (2014). Music interventions for the treatment of preoperative anxiety. J Consum Health Internet..

[38] Roomruangwong C, Tangwongchai S, Chokchainon A (2012). Preoperative anxiety among patients who were about to receive uterine dilatation and curettage. J Med Assoc Thai..

[39] Scott A (2004). Managing anxiety in ICU patients: the role of pre-operative information provision. Nurs Crit Care. avr.

[40] Lee K-C, Chao Y-H, Yiin J-J, Chiang P-Y, Chao Y-F. Effectiveness of different music-playing devices for reducing preoperative anxiety: a clinical control study. Int J Nurs Stud. 2011;48(10):1180-7. 10.1016/j.ijnurstu.2011.04.001 21565344

